# Aromatic amino acids play a harmonizing role in prostate cancer: A metabolomics-based cross-sectional study

**DOI:** 10.18502/ijrm.v19i8.9622

**Published:** 2021-09-09

**Authors:** Ziba Akbari, Roghayeh Taghipour Dijojin, Zahra Zamani, Reza Haji Hosseini, Mohammad Arjmand

**Affiliations:** ^1^Biochemistry Department, Metabolomics Lab, Pasture Institute of Iran, Tehran, Iran.; ^2^Biology Department, Payame Noor University, Tehran, Iran.

**Keywords:** Metabolomics, Prostate cancer, Aromatic amino acids, *${}^{1}$*H-NMR spectroscopy.

## Abstract

**Background:**

Prostate cancer (PCa) is a common health problem worldwide. The rate of this disease is likely to grow by 2021. PCa is a heterogeneous disorder, and various biochemical factors contribute to the development of this disease. The metabolome is the complete set of metabolites in a cell or biological sample and represents the downstream end product of the omics. Hence, to model PCa by computational systems biology, a preliminary metabolomics-based study was used to compare the metabolome profile pattern between healthy and PCa men.

**Objective:**

This study was carried out to highlight energy metabolism modification and assist the prognosis and treatment of disease with unique biomarkers.

**Materials and Methods:**

In this cross-sectional research, 26 men diagnosed with stage-III PCa and 26 healthy men with normal PSA levels were enrolled. Urine was analyzed with proton nuclear magnetic resonance (${}^{1}$H-NMR) spectroscopy, accompanied by the MetaboAnalyst web-based platform tool for metabolomics data analysis. Partial least squares regression discriminant analysis was applied to clarify the separation between the two groups. Outliers were documented and metabolites determined, followed by identifying biochemical pathways.

**Results:**

Our findings reveal that modifications in aromatic amino acid metabolism and some of their metabolites have a high potential for use as urinary PCa biomarkers. Tryptophan metabolism (p < 0.001), tyrosine metabolism (p < 0.001), phenylalanine, tyrosine and tryptophan biosynthesis (p < 0.001), phenylalanine metabolism (p = 0.01), ubiquinone and other terpenoid-quinone biosynthesis (p = 0.19), nitrogen metabolism (p = 0.21), and thiamine metabolism (p = 0.41) with Q${}^{2}$ (0.198) and R${}^{2}$ (0.583) were significantly altered.

**Conclusion:**

The discriminated metabolites and their pathways play an essential role in PCa causes and harmony.

## 1. Introduction

Prostate cancer (PCa) is a significant health problem, reported as the second most frequently diagnosed malignancy in men in 2018. The disease prevalence is one million cases per year. In Iran, PCa is the sixth most prominent cause of cancer death. More than 70,000 new cancer cases are reported every year in Iran (1), and PCa accounts for 5.3% (2).

The rate of cancer is expected to increase sharply in the next decade due to the increase in life expectancy and the aging in-country population. Scientists believe that the rate of PCa is likely to double by 2021. PCa is a heterogeneous disease; several factors such as aging, stress, genetic mutations (genetically), obesity, and lipid consumption contribute to the development of this disease (3). Environmental factors and lifestyle changes, such as weight changes, decreased physical activity, smoking, calcium levels, intake of dairy products, vitamin D levels, infection, and carcinogen environmental factors, could also contribute to causing PCa. The biopsy is the gold standard for diagnosing PCa and prostate-specific antigen (PSA) measurement and digital rectum examination (4). However, none of these methods are reliable (5), and the success rate of treatment significantly increases with the precise and early diagnosis of the disease (6).

Metabolomics is a fast-growing field in clinical research and provides new insight into various conditions and endpoints of omics cascade. Nowadays, metabolomics-based studies with proton nuclear magnetic resonance (${}^{1}$H-NMR) spectroscopy as analytical tools are the most applied methodologies in the metabolomics study of cancer (7, 8).

It has been reported that PCa cells display significantly altered metabolism compared to healthy ones in aggressive and recurring conditions and are associated with gene regulation of cancer-associated pathways. In tumor cells, energy requirements are met by reprogramming metabolic pathways to survive and proliferate. It has been stated that high levels of glucose are essential in the metabolism of many cancer types. Glucose is mainly consumed through the glycolysis pathway; however, fatty acid biosynthesis and amino acids such as glutamine and serine also generally increase in the cancer cell (9).

Therefore, in the present case-control retrospective study, we compared the metabolome profile pattern between healthy and PCa men by ${}^{1}$H-NMR spectroscopy. We determined the discriminated metabolites of both groups, highlighting the energy metabolism modification and facilitating data for more computational systems biology modeling approaches in the prognosis and treatment of this disease.

## 2. Materials and Methods

### Sample collection and preparation

In this cross-sectional study, 26 newly diagnosed PCa men were selected randomly based on their clinical-stage characteristics in mid-September 2018 at Bushehr General Hospital, Bushehr, Iran. PCa stage III was confirmed by a urologist's digital rectal examination and other related factors. In addition, 26 healthy men were also selected randomly based on their PSA level and were matched by age with the experimental group.

Serums and urine samples were collected in a preservative-free and sterile container. Serum was separated and kept at -80°C until assayed for total and free PSA. Urines samples were also centrifuged at 4000 rpm for 15 min at 4°C, and the supernatants were stored at -80°C until analyzed.

### PSA assay

Total and free PSA were assayed in duplicate by the Elisa colorimetric method. The plate was read at 450 nm with the reference wavelength at 630 nm in an Elisa reader.

### ${}^{1}$H-NMR experiment

Before analysis, the urine was thawed at room temperature and vortexed. 540 μL of urine was mixed with 60 μL of 100 mM phosphate buffer (pH 7.2) containing trimethylsilyl propionate (1 mM) as a chemical shift reference and imidazole (2 mM) as a pH indicator. Samples were then centrifuged for one min and transferred into NMR tubes.

The ${}^{1}$H-NMR spectrum was recorded on a Bruker Avance 500 MHz instrument. We used the universal pulse sequence protocol, one-dimensional nuclear overhauser effect spectroscopy (NOESY), with which spectra were obtained while suppressing the H${}_{2}$O signal through presaturation. The sequence started with a long or constant wave. It was accompanied by three high-power 90° pulses where a delay separated the first and second pulses, and the second and third pulses were separated by a mixing time. The free induction decay (FID) was obtained following the third 90° pulse with 300 scans.

### Spectral preprocessing

The spectrum of all samples was preprocessed using a custom-written ProMetab (v.3.3) function file, a metabolomics data processing device that changes raw Bruker NMR spectra into a format for multivariate study in the MATLAB environment. The region at 0-10 ppm of the spectra is segmented into bins of 0.005. The water peak at 4.7 ppm chemical shift was removed, and the resulting spectrum was normalized, followed by scaling with the Pareto scaling approach, employing the square root of the standard deviation as the scaling factor.

### Statistical multivariate metabolomics data analysis

MetaboAnalyst (v.4), an integrated web-based platform for a comprehensive analysis of quantitative metabolomics data, was used for multivariate analysis (10). Partial least square regression discriminant analysis (PLS-DA) used a supervised linear classification method to sharpen the separation between the two groups. Outliers were recorded, metabolites were determined using human metabolome databases (HMDB), and defined metabolites were used in the MetaboAnalyst pathway analysis to determine the discriminated biochemical pathways. The statistical p values from enrichment analysis are further adjusted for multiple testing.

### Ethical considerations

Written consent was obtained from all participants, and the design of this study was approved by Payeme-Noor University Ethics Committee (Code: IR.PNU.REC.1398.150).

## 3. Results

**Figure 1:**
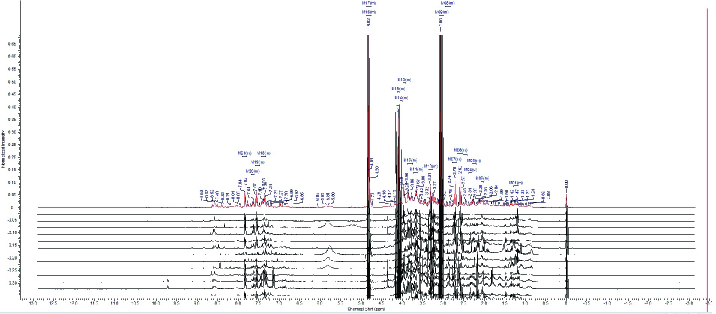
Superimposed ${}^{1}$H-NMR spectra of urine samples.

**Figure 2:**
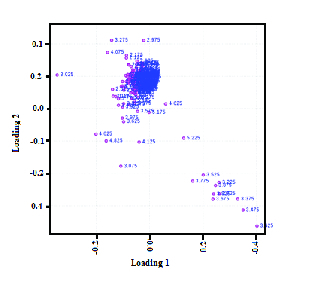
Loading plot dots denote the chemical shift bin.

Table I presents the demographic characteristics and clinical characterization of both groups. The results from the pathway analysis demonstrated that several metabolic pathways were affected by PCa. The comparison of the spectra patterns in men with PCa and the healthy group is exhibited in Figure 1. Outliers were separated by using the loading plot (Figure 2).

**Figure 3:**
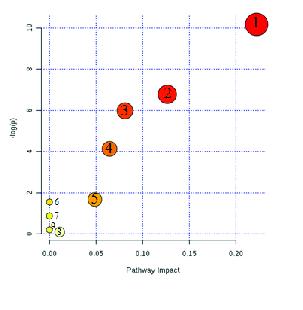
The degree of centrality of chemical pathways shows the number of links incident upon a node. (1) Tryptophan metabolism, (2) Tyrosine metabolism, (3) Phenylalanine, tyrosine, and tryptophan biosynthesis, (4) Phenylalanine metabolism, (5) Ubiquinone and other terpenoids-quinone biosynthesis, (6) Nitrogen metabolism, (7) Thiamine metabolism, (8) Aminoacyl-tRNA biosynthesis and (9) Porphyrin and chlorophyll metabolism.

Moreover, the topology map of altered biochemical pathways in the test and control groups is shown in Figure 3. Degree centrality was defined as the number of links that occurred upon a node. In our investigation, three major biochemical pathways with a p-value < 0.05 were used in our discussion, while other pathways were kept apart (Table II).

Cross-validation (10fold-CV) was performed to validate our results. The Q2 value, which indicates the validity of the PLS-DA discrimination, was 0.198, whereas R2 was 0.58322 with an accuracy of 0.7954 in component 3.

**Table 1:** Demographic and clinical characterization of the healthy and experimental groups


		**PCa men**	**Healthy**
**Mean age (yr)**			
	**40-50**	46.77	46.08
	**51-60**	55.47	55
**Total PSA (level ± SD)**			
	**Normal range 0.0-4.5 ng/mL**	- 1.11 ± 0.07
	**> 4.5 ng/mL**	39.82 ± 2.6	-
	**Free PSA ratio**	6.8 ± 2	-
Experimental and healthy samples were obtained from men aged 40-60 according to the above data. PSA: Prostate-specific antigen, compression of total and free PSA ratio in both groups also described			

**Table 2:** Discriminated metabolites and their pathways from pathway analysis


**Pathway**	**Metabolites**	**Total**	**Hits**	**p-value**	**FDR**	**Impact**
**Tryptophan metabolism**	5-N-Acetylserotonin; Methoxytryptamine; 5-Hydroxyindoleacetate; Melatonin; Indole-3-acetamide; Indole-3-acetate; Anthranilate; 3-Hydroxyanthranilate; Xanthurenic acid	79	9	< 0.001	0.000306	0.22
**Tyrosine metabolism**	L-Tyrosine; 4-Hydroxyphenylpyruvate; Phenol; Formamide; Triiodothyronine; Hydroquinonecarboxylic acid; gentisate; 3,4Dihydroxyphenylpropanoate	76	7	< 0.001	0.00454	0.13
**Phenylalanine, tyrosine, and tryptophan biosynthesis**	3,4-Dihydroxybenzoic acid; Anthranilic acid; 4-Hydroxyphenylpyruvate; L-Tyrosine	27	4	< 0.001	0.00674	0.08
**Phenylalanine metabolism**	Phenethylamine; Phenylacetic acid; 2-Hydroxyphenylacetate L-Tyrosine	45	4	0.01	0.0320	0.06
**Ubiquinone and another terpenoid-quinone biosynthesis**	3-(4-Hydroxyphenyl) pyruvate; L-Tyrosine	36	2	0.19	0.0187	0.05
**Nitrogen metabolism**	Anthranilate; L-Tyrosine	39	2	0.21	1.55	0.00
**Thiamine metabolism**	L-Tyrosine	24	1	0.41	0.0878	0.00
**Aminoacyl-tRNA biosynthesis**	L-Tyrosine	75	1	0.81	0.0203	0.00
**Porphyrin and chlorophyll metabolism**	Bilirubin	104	1	0.90	0.0984	0.01
Total: Total number of compounds in the pathway, Hits: Matched number from the user uploaded data, P-value: Original p-value calculated from the enrichment analysis, FDR: P-value adjusted using false discovery rate, Impact: Pathway impact value calculated from pathway topology analysis						

## 4. Discussion

The prostate displays an exceptional metabolism that changes during primary neoplasia to destructive PCa (11). Although several biochemical studies have been carried out on PCa, very few systematic reviews have been performed to discover the metabolite pattern alterations in the urine of these men (12). So, in need of raw systematic metabolome data for PCa modeling, we compared the urinary metabolome profile of PCa men with that of healthy men for further modeling of PCa by systems biology methods.

Urine is considered the primary source of released PCa biomarkers (13). Change in sarcosine is reported as one of the essential factors for identifying PCa (14). It has been reported that the urinary excretion of isoleucine in PCa men is considered one of the most altered parameters in PCa (15). Although alternation of aromatic amino acid pathways causes many types of cancers and disorders, only a few investigations have concentrated on the importance of these pathways in cancer. Based on our results, the present metabolomics pilot study suggested that alterations of aromatic amino acid pathways (i.e., tryptophan tyrosine and phenylalanine metabolism) and some of their metabolites have a high potential for use as urinary PCa biomarkers.

It has been reported that tryptophan metabolism is changed in PCa (16). According to the enrichment analysis (p < 0.05), the most significant changes in our study were linked to tryptophan metabolism. This pathway is associated with cancer and the immune system. Our results showed acetylserotonin, methoxytryptamine, hydroxyindoleacetate, melatonin, indoleacetamide, indoleacetate, anthranilate, hydroxyanthranilate, and xanthurenic acid metabolites were altered in this pathway.

It has been stated that serotonin, which is produced from tryptophan in prostate cells, had a critical role in the growth, inhibition, and induction of apoptosis in tumor prostate cells (17). Serotonin is also linked to some metabolites such as N-Acetyl serotonin, melatonin, and 5-hydroxyindoleacetate as a precursor. Therefore, any alteration in serotonin can affect these metabolites. In our study, melatonin was one of the altered metabolites in tryptophan metabolism. According to the results of Hevia and colleagues, melatonin can be detected easily in PCa cells. They also showed a significant relationship between cellular melatonin uptake and anti-proliferative melatonin activity in some prostatic cancer cells (18).

Similarly, in other studies, it has been demonstrated that that melatonin can inhibit the growth of PCa cells by affecting the androgen-sensitive and insensitive epithelium of PCa cells (19). Also, glucose uptake in glycolysis, the Krebs cycle, and the pentose phosphate pathway is limited by melatonin in PCa cells (18). Any alteration in cellular melatonin could change the serum or urine concentration, which aligns with our study. Indole compounds are aromatic heterocyclic compounds that are involved in inhibiting cell proliferation and inducing apoptosis in cells. Furthermore, Indoles can inhibit tumor formation in the prostate. The phosphatidylinositol 3-kinase (PI3K)/AKT pathway has a positive effect on regulating the balance between cell viability and apoptosis in tumor cells, and some studies reported that indole compounds suppress the activation of the PI3K/AKT pathway in prostate carcinogenesis (20).

**Figure 4:**
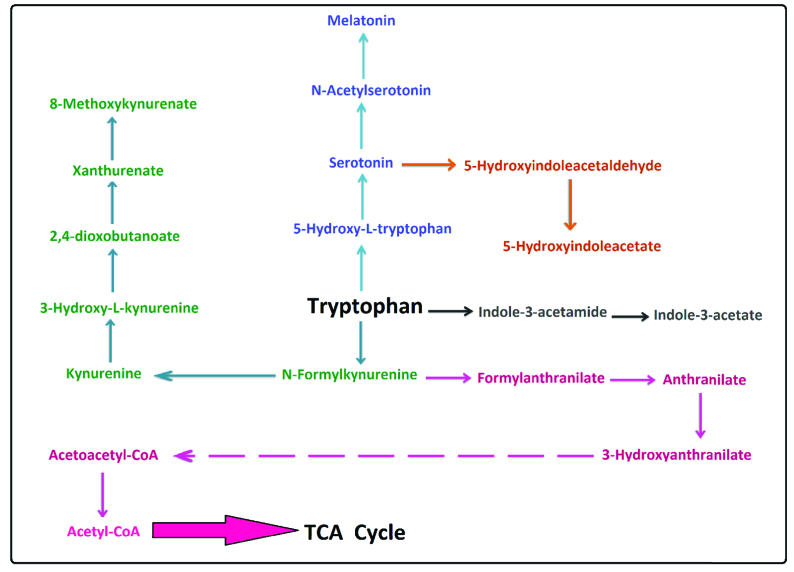
Overview of tryptophan metabolism changes in PCa men.

It has been exhibited that some kynurenine pathway metabolites such as L-tryptophan, kynurenine, anthranilate, and indolelactate were altered in the serum of the PCa men they studied (16). Our findings are in line with a recent study, which indicated that the urinary concentration of indoleacetamide and indoleacetate in tryptophan metabolism was altered in PCa males (Figure 4) (17).

Tryptophan metabolism is linked to TCA cycles and ATP production in the phosphorylation oxidate pathway by some metabolites like anthranilate and 3-hydroxy anthranilates. Changes in anthranilate, which have been reported in tryptophan metabolism and phenylalanine tyrosine and tryptophan biosynthesis (16), are supported by our findings.

**Figure 5:**
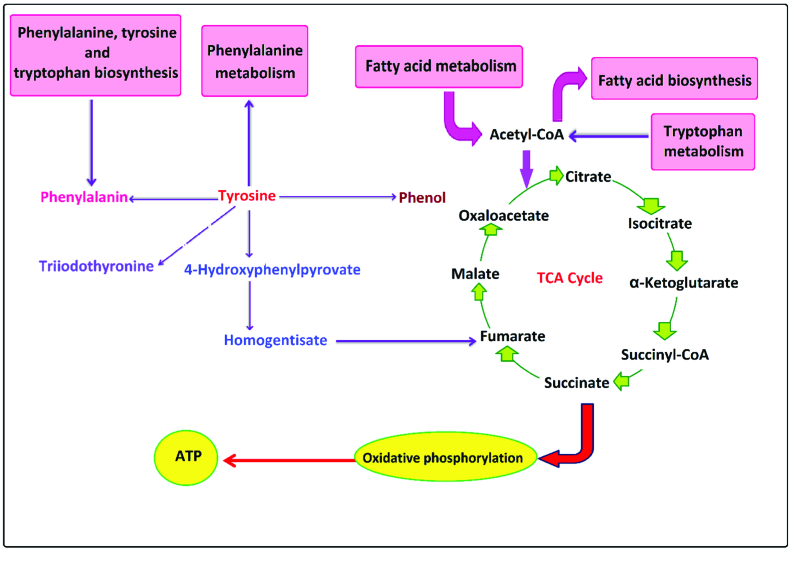
Overview of tyrosine metabolism changes in PCa men.

Furthermore, our data indicate that the tyrosine metabolic pathway was changed in PCa, and metabolites such as L-tyrosine, hydroxyphenylpyruvate, phenol, formamides, triiodothyronine, hydroquinone carboxylic acid, and dihydroxy phenyl propanoate were altered (Figure 5). Our findings showed that tyrosine was a metabolite that was changed, not in tyrosine metabolism, but phenylalanine, tyrosine, tryptophan biosynthesis, and the phenylalanine metabolic pathways. Tyrosine is catalyzed to 4-hydroxyphenylpyruvate and converted into homogentisic acid by the 4-hydroxyphenylpyruvate dioxygenase (HPD) enzyme. Previous studies have shown that HPD can be highly expressed in lung carcinoma. Suppression of HPD expression is related to a decrease in tumor cell proliferation by altering some pathways such as pentose phosphate, RNA biosynthesis, and reactive oxygen species (20).

Tyrosine is involved in regulating protein acetylation, histone modification, and acetyl-CoA gene expression (20). Tyrosine and 4-hydroxyphenylpyruvate are linked to TCA and energy production by conversion to homogentisate and fumarate sequentially. Our findings suggest that PCa cells depend on TCA cycles and fatty acid metabolism for ATP production. It has been hypothesized that early PCa did not rely on glycolysis and the Warburg effect in the men (21). However, it dominates in these cells, so both ATP and acetyl-CoA are provided by fatty acid oxidation. Thyroid hormones have shown different effects in PCa cells, depending on the thyroid hormone involved (T3 or T4) (22). Lehrer et al*.* reported that triiodothyronine was increased in men with PCa and showed the mitogenic effects on prostatic cancer cells. They suggested the hypothesis that triiodothyronine might be a valuable biomarker for PCa (23). An association of elevated serum triiodothyronine with an increased risk of recurrent PCa has also been reported. Similarly, higher free T4 is associated with an increased risk of PCa (24). Our findings also support these hypotheses.

Phenolic compounds are known as anti-cancer and anti-metastatic agents. Phenolic compounds inhibit the initiation and progression of several signaling pathways in various types of cancer (25). Our results also reveal that the urinary content of phenol and triiodothyronine changed in PCa men.

3,4-dihydroxybenzoic acid in phenylalanine tyrosine and tryptophan biosynthesis is a protective agent against cardiovascular diseases and carcinoma due to its antioxidant activity. A few reports have shown that 3,4-dihydroxybenzoic acid can change colorectal cancer. Canarium schweinfurthii Engl oil, which contains dihydroxybenzoic acid polyphenol, has been shown to possess PCa chemopreventative effects (26).

In this study, the urinary level of phenethylamine, L-tyrosine, phenylacetic acid, and 2-hydroxyphenylacetate were altered in the phenylalanine metabolic pathway. Phenylacetic acid is a decomposed protein. It has high antitumor activity and plays a vital role in the control and differentiation of cell growth. It has been reported that it is a potent factor for treating human tumors (27). Moreover, it has been shown that an increase in plasma concentrations of phenylacetic acid in end-stage renal failure leading to the inhibition of iNOS expression (28). Phenylethylamine is a neurotransmitter, and it is biosynthesized from tryptophan by enzymatic decarboxylation (29). There is an increase in the urinary concentration of phenylethylamine in people with autism and minimal brain dysfunction (30). 2-hydroxyphenylacetate is present in all biofluids and even in the prostate, kidney, and bladder tissues and is associated with colorectal and lung cancer (31).

The concentration of 2-hydroxyphenylacetic acid was altered in our study. 2-hydroxyphenylacetic acid is a substrate of the enzyme oxidoreductases. Recently, the potential impact of the combined inhibition of 3a-oxidoreductases and 5a-reductases on PCa it has been reported (32). Our findings also reveal that the urinary level of phenylalanine, L-tyrosine, phenylacetic acid, and 2-hydroxyphenylacetate metabolites were altered in the phenylalanine metabolic pathway.

## 5. Conclusion

We conclude that the discriminated metabolites in aromatic amino acid metabolism play an essential role in causing PCa and its metabolic harmony. Further systematic approaches are required to complete and validate this notion in computational systems biological modeling of PCa, to help work towards offering unique biomarkers for clinical practice.

##  Conflict of Interest

The authors have no conflict of interest to declare.
